# Studying variability in human brain aging in a population-based German cohort—rationale and design of 1000BRAINS

**DOI:** 10.3389/fnagi.2014.00149

**Published:** 2014-07-14

**Authors:** Svenja Caspers, Susanne Moebus, Silke Lux, Noreen Pundt, Holger Schütz, Thomas W. Mühleisen, Vincent Gras, Simon B. Eickhoff, Sandro Romanzetti, Tony Stöcker, Rüdiger Stirnberg, Mehmet E. Kirlangic, Martina Minnerop, Peter Pieperhoff, Ulrich Mödder, Samir Das, Alan C. Evans, Karl-Heinz Jöckel, Raimund Erbel, Sven Cichon, Markus M. Nöthen, Dieter Sturma, Andreas Bauer, N. Jon Shah, Karl Zilles, Katrin Amunts

**Affiliations:** ^1^Institute of Neuroscience and Medicine (INM-1, INM-2, INM-4, INM-8), Research Centre JülichJülich, Germany; ^2^Institute of Medical Informatics, Biometry and Epidemiology, University of Duisburg-EssenEssen, Germany; ^3^Department of Genomics, Life & Brain Center, University of BonnBonn, Germany; ^4^Institute of Human Genetics, University of BonnBonn, Germany; ^5^Institute for Clinical Neuroscience and Medical Psychology, University of DüsseldorfDüsseldorf, Germany; ^6^McConnell Brain Imaging Center, Montreal Neurological Institute, McGill UniversityMontreal, QC, Canada; ^7^Department of Cardiology, University of Duisburg-EssenEssen, Germany; ^8^Division of Medical Genetics, Department of Biomedicine, University of BaselBasel, Switzerland; ^9^Institute for Science and Ethics, University of BonnBonn, Germany; ^10^Department of Neurology, Heinrich-Heine-University DüsseldorfDüsseldorf, Germany; ^11^JARA-BRAIN, Jülich-Aachen Research AllianceJülich, Germany; ^12^Department of Neurology, RWTH Aachen UniversityAachen, Germany; ^13^Department of Psychiatry, Psychotherapy, and Psychosomatics, RWTH Aachen UniversityAachen, Germany; ^14^C. and O. Vogt Institute for Brain Research, Heinrich-Heine-University DüsseldorfDüsseldorf, Germany

**Keywords:** cohort, connectivity, Heinz Nixdorf Recall Study, resting-state, imaging genetics, variability, aging, elderly

## Abstract

The ongoing 1000 brains study (1000BRAINS) is an epidemiological and neuroscientific investigation of structural and functional variability in the human brain during aging. The two recruitment sources are the 10-year follow-up cohort of the German Heinz Nixdorf Recall (HNR) Study, and the HNR MultiGeneration Study cohort, which comprises spouses and offspring of HNR subjects. The HNR is a longitudinal epidemiological investigation of cardiovascular risk factors, with a comprehensive collection of clinical, laboratory, socioeconomic, and environmental data from population-based subjects aged 45–75 years on inclusion. HNR subjects underwent detailed assessments in 2000, 2006, and 2011, and completed annual postal questionnaires on health status. 1000BRAINS accesses these HNR data and applies a separate protocol comprising: neuropsychological tests of attention, memory, executive functions and language; examination of motor skills; ratings of personality, life quality, mood and daily activities; analysis of laboratory and genetic data; and state-of-the-art magnetic resonance imaging (MRI, 3 Tesla) of the brain. The latter includes (i) 3D-T1- and 3D-T2-weighted scans for structural analyses and myelin mapping; (ii) three diffusion imaging sequences optimized for diffusion tensor imaging, high-angular resolution diffusion imaging for detailed fiber tracking and for diffusion kurtosis imaging; (iii) resting-state and task-based functional MRI; and (iv) fluid-attenuated inversion recovery and MR angiography for the detection of vascular lesions and the mapping of white matter lesions. The unique design of 1000BRAINS allows: (i) comprehensive investigation of various influences including genetics, environment and health status on variability in brain structure and function during aging; and (ii) identification of the impact of selected influencing factors on specific cognitive subsystems and their anatomical correlates.

## Introduction

The process of aging varies widely between individuals, particularly in relation to cognitive abilities, motor skills, and changes in brain structure and function (for review e.g., Stern, 2009, 2012; Bartrés-Faz and Arenaza-Urquijo, 2011). The aging spectrum ranges between accelerated aging and the preservation of high levels of physical fitness and mental agility into old age (Tucker and Stern, 2011). Appraisal of the implications of aging also varies widely between individuals (for review e.g., Jeste et al., 2010), and the concept of “successful aging” has been the focus of extensive research (for review e.g., Vaillant and Mukamal, 2001; Depp and Jeste, 2006).

Studies comparing elderly and young subjects have generated evidence for correlations between changes in physical and mental performance and changes in brain morphology and activity across various cognitive domains (for review e.g., Cabeza, 2002; Grady, 2008; Spreng et al., 2010; Eyler et al., 2011; Turner and Spreng, 2012). Changes in motor performance, attention, (working) memory, executive control, and processing speed are of particular interest, since deterioration in these domains has a detrimental impact on the activities of daily living (for review e.g., Park and Reuter-Lorenz, 2009; Reuter-Lorenz and Park, 2010). In addition to a decline in the integrity of the musculoskeletal system, central motor control and execution are adversely affected by aging, leading to a decrease in the capacity to plan and coordinate complex movements (for review e.g., Krampe, 2002; Seidler et al., 2010). This in turn increases the risk of falls (for review e.g., Bloem et al., 2003).

At the time of writing, the factors which influence cognitive reserve capacity remain largely unknown (Ward, 2006). Previous studies have suggested the existence of compensatory mechanisms that maintain stable performance over time, e.g., by increasing functional connectivity between brain areas. In contrast, other studies of aging reported only limited or no compensatory effects at all (for review e.g., Raz and Rodrigue, 2006; Goh and Park, 2009; Steffener and Stern, 2012). Few data are available concerning differences in the compensatory capacity of the human brain. However, this capacity is likely to be influenced by a range of currently unknown factors (Tucker and Stern, 2011). Delineating structural and functional brain plasticity and adaptability during aging in elderly is rendered even more challenging. Structural and functional brain changes have been found throughout the life-span, from early to late adulthood (e.g., Pieperhoff et al., 2008; Mowinckel et al., 2012). For this reason, identification of potential influencing factors and their interactions with brain function or behavior in elderly is insufficient. Instead, research must also determine why the presence or absence of a given factor may result in completely different brain phenotypes in different subjects. At present, the respective interplay between genetic and environmental factors remains unclear.

Available research therefore indicates the existence of considerable interindividual variability in aging as a result of an interplay between numerous external (e.g., environmental, psychoscocial, socioeconomic) and internal (e.g., genetic, physiological) factors. Characterization of how the range of observable phenotypes in the aging brain is influenced by or relates to these factors and any interactions of them may therefore facilitate disentanglement of the underlying processes of aging. To represent variability in the aging process of the human brain, investigation of a large cohort of elderly subjects from the general population is required. Furthermore, the research approach must involve comprehensive analysis of structural and functional brain phenotypes and related motor and cognitive performance, as well the investigation of a wide range of factors with a potential role in the observed behavioral, structural, and functional variability.

The ongoing 1000BRAINS study was designed to address these challenges. The aims of 1000BRAINS are to: (i) identify age-dependent changes in the elderly human brain; (ii) characterize the phenotypical variability observed during brain aging; and (iii) investigate the causes of intraindividual variability in the process of aging. The 1000BRAINS cohort is being drawn from two sources: (i) the longitudinal population-based German Heinz Nixdorf Recall (HNR) Study (Schmermund et al., 2002; Erbel et al., 2012), whose dataset represents a valuable resource for aging research; and (ii) the HNR MultiGenerationStudy, which is comprised of the spouses and offspring of HNR subjects. In addition to use of the HNR data, 1000BRAINS involves state-of-the-art neuroimaging methods and molecular genetic techniques. Neuroimaging represents a powerful tool for detecting even subtle changes in brain morphology and function during brain aging in elderly subjects, while increasing understanding of the human genome and the use of innovative molecular genetic techniques may provide insights into the genetic processes which underlie variation in age-related brain phenotypes.

The aim of the present report is to describe the cohort, the protocol, and the magnetic resonance imaging (MRI) methods of the 1000BRAINS study.

## Study design

1000BRAINS is a cohort study. A total of 1000 subjects will be recruited from two existing cohorts and assessed in order to characterize interindividual variability in brain aging. A sample size of *n* = 1000 was selected as determination of correlations between variability in brain structure and function with increasing age and environmental and genetic risk factors requires a large cohort. This sample size is comparable to those of previous large-scale neuroimaging studies in Europe and the US (Mueller et al., 2005; Ikram et al., 2012; Van Essen et al., 2012), which achieved a uniform distribution of subjects in different age groups (Button et al., 2013). An adequate sample size is also required to detect genetic influences with small effect sizes in polygenic phenotypes (Stein et al., 2012). An advantage of 1000BRAINS is that for all levels of data the applied methods and testing procedures are consistent across all subjects. This optimizes their reliability and generates homogeneous data in a large sample of subjects.

## Recruitment sources

The aim of the HNR study is to investigate correlations between lifestyle-, social-, and environmental conditions and risk factors for (i) atherosclerosis (in particular coronary calcification); and (ii) myocardial infarction (Recall: Risk factors evaluation of coronary calcium and lifestyle). The baseline HNR cohort was recruited from a sample that had been drawn at random from the citizen registries of the cities of Essen, Bochum, and Mühlheim. These cities are located in the Ruhr area, a metropolitan region with a population of >11 million. Recruitment for the HNR study commenced in 2000 with the inclusion of 4814 subjects aged 45–75 years (50.2% female; baseline response 56%). The 5 year follow-up assessments commenced in 2006 (response: 90%). The 10 year follow-up assessments commenced in June 2011 and are ongoing at the time of writing. The extensive HNR study protocol comprises standardized physical examinations, questionnaires, and interviews at baseline and at 5- and 10-year follow-up. In addition, subjects have completed annual postal questionnaires on health status.

**Figure 1:**
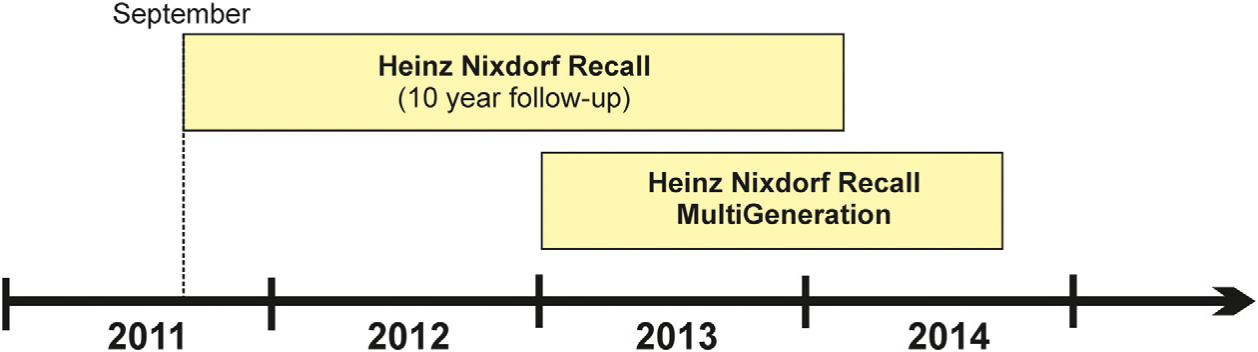
**Timeline of the 1000BRAINS study which commenced in September 2011.** The figure is subdivided into the two recruitment sources, i.e., the cohorts of the Heinz Nixdorf Recall Study and the Heinz Nixdorf MultiGeneration study.

Subjects attending the 10-year HNR follow-up assessment (estimated total number: 3500 subjects) are asked to participate in 1000BRAINS. To date, 71% of HNR subjects have been willing to participate in 1000BRAINS, while a further 7% were undecided at the time of first contact. At the HNR study center in Essen, unwillingness to participate in 1000BRAINS has been attributed to: (i) unwillingness to undergo further extensive examinations, which involve a journey of 100–120 km (90–120 min driving time, depending on place of residence and traffic) to the 1000BRAINS research site at the Research Centre Jülich; and (ii) the presence of debilitating major illnesses such as cancer or cardiorespiratory insufficiency. Subjects who are willing to participate in 1000BRAINS are screened for eligibility according to specified exclusion criteria (see section Study Protocol). To date, 23% of all HNR 10-year follow up subjects have been included in 1000BRAINS. The first 1000BRAINS assessments were completed in September 2011 (Figure 1).

The HNR MultiGeneration Study commenced in January 2013. In this study, the spouses and offspring of the baseline HNR subjects are being recruited in order to investigate cardiovascular risk factor burden and the related incidence of cardiovascular disease within families. Approximately 5500 individuals are eligible for this sample. A detailed description of this study will be provided elsewhere. 1000BRAINS commenced its recruitment of HNR MultiGeneration Study subjects in April 2013. Inclusion of these subjects in 1000BRAINS in particular enables investigation of both: (i) younger subjects (i.e., <55 years); and (ii) the influence of inherited genetic factors and their interaction with environmental factors on brain aging phenotypes.

As of December 31st 2013, a total of 653 subjects from these two HNR cohorts had been examined in accordance with the 1000BRAINS study protocol.

## Study protocol

To enable comprehensive investigation of the aging of the human brain and its interindividual variability in aging, 1000BRAINS applies state-of-the-art neuroscientific methods and techniques. These include: (i) a detailed MRI protocol for studying the structure and function of the aging brain; and (ii) neuropsychological tests of a wide range of cognitive functions and aspects of motor performance. Psychological variables, such as personality traits and the presence or absence of a subjective sense of well-being, are measured using standardized questionnaires. Laboratory, genetic, epigenetic, and expression analyses are performed to identify whether specific blood metabolites, genetic polymorphisms, epigenetic signatures or expression profiles influence brain aging phenotypes. Details of each aspect of the protocol are provided below. The study protocol was approved by the ethics committee of the University of Duisburg-Essen. The study procedures comply with the Declaration of Helsinki. Strict internal and external quality assurance protocols are applied during all 1000BRAINS study procedures.

### Participants

#### Exclusion criteria

Study exclusion is conditional on eligibility for MR measurements. The MR exclusion criteria used in 1000BRAINS are consistent with standard ethical and MR safety guidelines for the MR examination of healthy subjects for an exclusively scientific purpose. HNR subjects are excluded from 1000BRAINS if any of the following are present: coronary artery stents; cardiac pacemakers; or surgical implants or prostheses in the trunk or head. This is a necessary precaution, since during the scanning procedure, the brain, trunk, and head are exposed to a strong static magnetic field, pulsed magnetic field gradients, and radiofrequency fields. Further exclusion criteria are: claustrophobia; a history of neurosurgery; and the presence of tattoos or permanent make-up on the head. Dental implants and bridges are relative contraindications. In such cases, scanning is discontinued if the respective object produces artifacts in the brain images during the localizer sequence stage.

Potential 1000BRAINS participants are informed of the aims and procedures of the study at the HNR study center in Essen by trained study personnel. During this discussion, they are also screened for MR contraindications using a standardized interview. Willing and eligible subjects are then asked to sign the informed consent form, and an appointment for the 1000BRAINS examination in Jülich is arranged. An uncertain surgical history is clarified by requesting a report from the hospital at which surgery was performed. The report is then evaluated by an experienced physician and an MR physicist responsible for MR safety at the Research Centre Jülich. These experts then make the final decision regarding eligibility for study participation. This approach enables avoidance of the general exclusion of subjects who have undergone surgery and thus maximizes recruitment.

#### Quality assurance

All examinations and testing procedures are conducted in accordance with predefined guidelines, as detailed in the respective standard operating procedures. MRI is performed by trained technical personnel. Neuropsychological and motor testing are performed by raters (experienced study nurse as well as assistants at bachelors and masters level). The raters are trained by an experienced neuropsychologist for the neuropsychological testing and by an experienced neurologist for the motor testing and have then undergone a period of supervision before being allowed to assess subjects independently. All raters are continuously supervised to assure constant quality of the testing. Quality control is further assured by the video taping of all tests for evaluation of the neuropsychological testing by an experienced neuropsychologist and of the motor testing by an experienced neurologist.

The MR data of all subjects are reviewed by an experienced radiologist in order to: detect pathological findings, in which case the subject is excluded from further analysis; and (ii) report on any incidental findings requiring medical attention (see below).

An established data base system is used for data storage and management (see below). Double data entry is performed to optimize quality control.

#### Incidental findings

Since the MRI data of the 1000BRAINS subjects are reviewed by an experienced radiologist, incidental findings may arise. During the informed consent procedure and prior to inclusion, all potential 1000BRAINS subjects must agree to be informed about any incidental finding requiring medical attention. Subjects who refuse this will be excluded from the study. This strategy is in accordance with current guidelines (e.g., http://www.bioethics.gov), and with standard procedures implemented in on-going brain imaging studies (e.g., Study of Health in Pomerania (SHIP), Hegenscheid et al., 2009, 2013). If the radiologist reports an incidental finding, the case is discussed by the members of an internal 1000BRAINS disease advisory board, which is comprised of physicians and epidemiologists. The board decides whether the subject should be informed. If indicated, the respective subject is then informed of the finding by a study physician, and requested to consult their primary care physician for further evaluation and management. This procedure is regularly discussed with neuroethicists.

Conditions requiring urgent medical evaluation include brain tumors, arteriovenous malformations, aneurysms, hemorrhages, inflammation, and acute stroke. Conditions warranting routine medical referral include post stroke status or mucosal thickening within the sinus and the mastoid cells. Age-related brain changes may also be detected. These include white matter lesions, which are a frequent radiological finding. In accordance with previous studies (e.g., SHIP, Hegenscheid et al., 2009), such age-related findings will not be reported to the subject.

### Measurements and testing

#### Magnetic Resonance Imaging (MRI)

**Figure 2:**
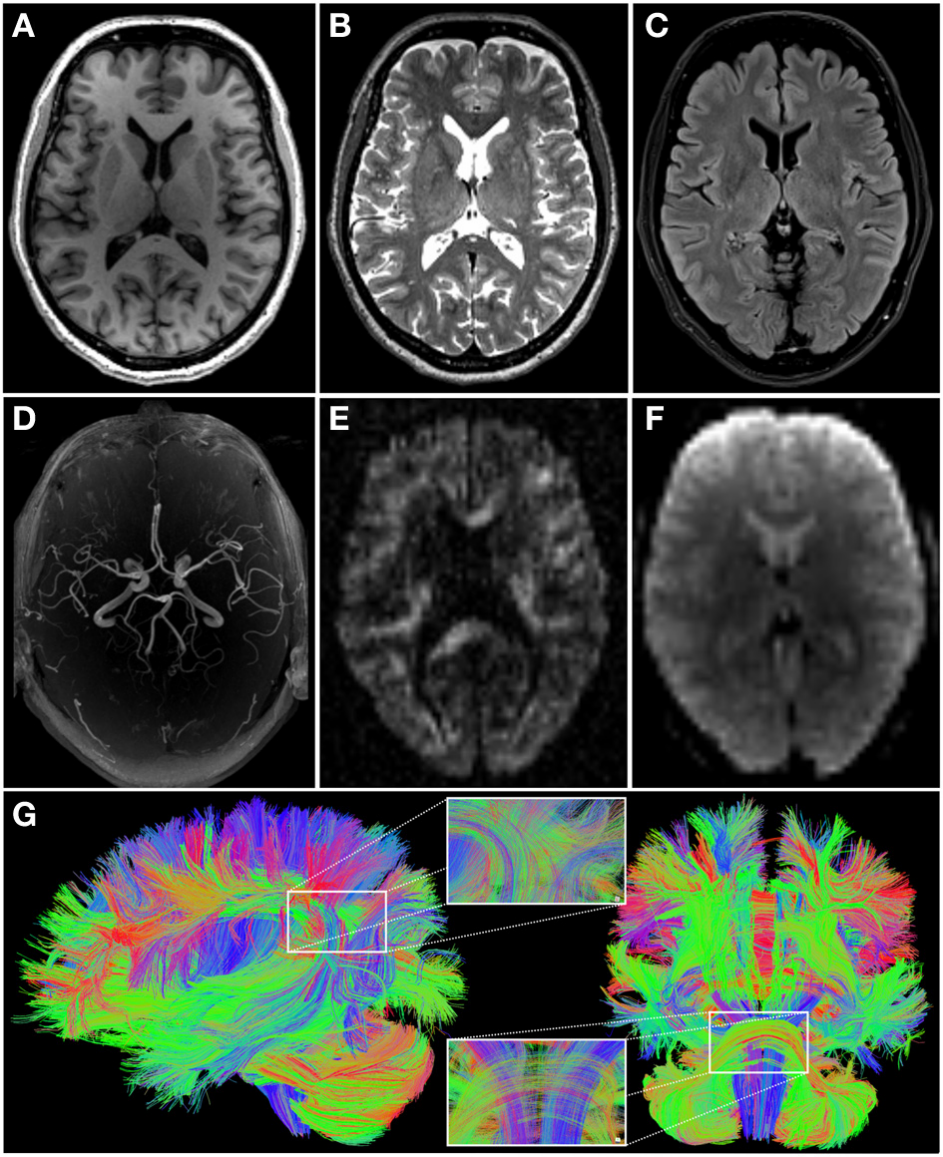
**Images illustrating the different modalities of magnetic resonance imaging data acquired in 1000BRAINS (cf. Table 1). (A)** T1-weighted; **(B)** T2-weighted; **(C)** fluid attenuated inversion recovery (FLAIR); **(D)** axial maximum intensity projection (MIP) of time-of-flight angiography; **(E)** high angular resolution diffusion imaging (HARDI, one out of 120 diffusion weighted images); **(F)** functional and resting-state echo-planar image (EPI). **(G)** Additionally, exemplary reconstruction of whole brain fiber architecture from HARDI data [as depicted in **(E)**], using TrackVis software (http://www.trackvis.org), from the lateral (left) and anterior (right) views. Inlays show enlargements of the fiber architecture within the inferior parietal cortex (top) and the pons and brainstem (bottom). Color coding of fiber directions in accordance with common conventions (blue: top—bottom; red: left—right; green: rostral—caudal).

MRI is carried out on a 3 Tesla MR scanner (Tim-TRIO, Siemens Medical Systems, Erlangen, Germany). A range of sequences have been chosen to maximize insight into the structure and function of the brain in a large number of subjects within a reasonable time-frame (Table 1, Figure 2).

**Table 1:** **Overview over the MRI sequences used in 1000BRAINS with relevant sequence parameters**.

**Name**	**Sequence parameters**
T1 (3D-MPRAGE)	176 slices, *TR* = 2.25 s, *TE* = 3.03 ms, *TI* = 900 ms, FoV = 256 × 256 mm^2^, flip angle = 9°, voxel resolution = 1 × 1 × 1 mm^3^
T2 (3D-SPACE)	176 slices, *TR* = 3.2 s, *TE* = 417 ms, FoV = 256 × 256 mm^2^, voxel resolution = 1 × 1 × 1 mm^3^
DTI (30 directions)	EPI, *TR* = 7.8 s, *TE* = 83 ms, 4 b0-images (interleaved), 30 images with *b* = 1000 s/mm^2^, voxel resolution = 2 × 2 × 2 mm^3^
DTI (60 directions, HARDI subset)	EPI, *TR* = 6.3 s, *TE* = 81 ms, 7 b0-images (interleaved), 60 images with *b* = 1000 s/mm^2^, voxel resolution = 2.4 × 2.4 × 2.4 mm^3^
HARDI (120 directions)	EPI, *TR* = 8 s, *TE* = 112 ms, 13 b0-images (interleaved), 120 images with *b* = 2700 s/mm^2^, voxel resolution = 2.4 × 2.4 × 2.4 mm^3^
Functional and resting-state MRI	EPI, 36 slices, *TR* = 2.2 s, *TE* = 30 ms, FoV = 200 × 200 mm^2^, flip angle = 90°, voxel resolution = 3.1 × 3.1 × 3.1 mm^3^
T2 (FLAIR)	25 slices, *TR* = 9 s, *TE* = 100 ms, FoV = 220 × 220 mm^2^, flip angle = 150°, voxel resolution = 0.9 × 0.9 × 4 mm^3^
Angiography (ToF)	40 slices, *TR* = 22 ms, *TE* = 3.86 ms, voxel resolution = 0.7 × 0.5 × 0.7 mm^3^

DTI, diffusion tensor imaging; EPI, gradient-echo echoplanar imaging; FLAIR, fluid-attenuated inversion recovery; FoV, field of view; HARDI, high angular resolution diffusion imaging; MPRAGE, magnetization prepared rapid acquisition gradient echo; SPACE, sampling perfection with application optimized contrasts using different flip angle evolution; TE, echo time; TI, inversion time; ToF, time of flight; TR, repetition time. T1, T2, weighting of the applied MRI sequence.

Structural scans include an anatomical 3D T1-weighted MPRAGE sequence, a 3D T2-weighted SPACE sequence, a clinical T2-weighted FLAIR sequence, MR-angiography of the basal brain arteries without contrast agents using a Time of Flight (ToF) sequence, and 3 different diffusion-weighted sequences (30/60/120 diffusion directions). The latter are optimized for the following processing strategies: (i) diffusion tensor imaging (DTI), with emphasis on the analysis of fractional anisotropy as a measure of white matter integrity; (ii) high angular resolution diffusion imaging (HARDI) data to allow tractography-based reconstructions of white matter fiber paths (Figure 2); and (iii) diffusion kurtosis imaging as a measure of white matter integrity under non-Gaussian assumptions of water diffusion in the brain.

Functional scans include a resting-state scan of 11 min duration (eyes closed, light switched off, instruction: let the mind wander without thinking of anything in particular), and functional imaging paradigms for a range of functional systems. The latter include action processing, working memory, stimulus-response mapping, and quantifier processing. Using optimized sequence parameters and parallel imaging, the total scanning time is approximately 75–90 min, depending on the functional imaging paradigm.

**Figure 3:**
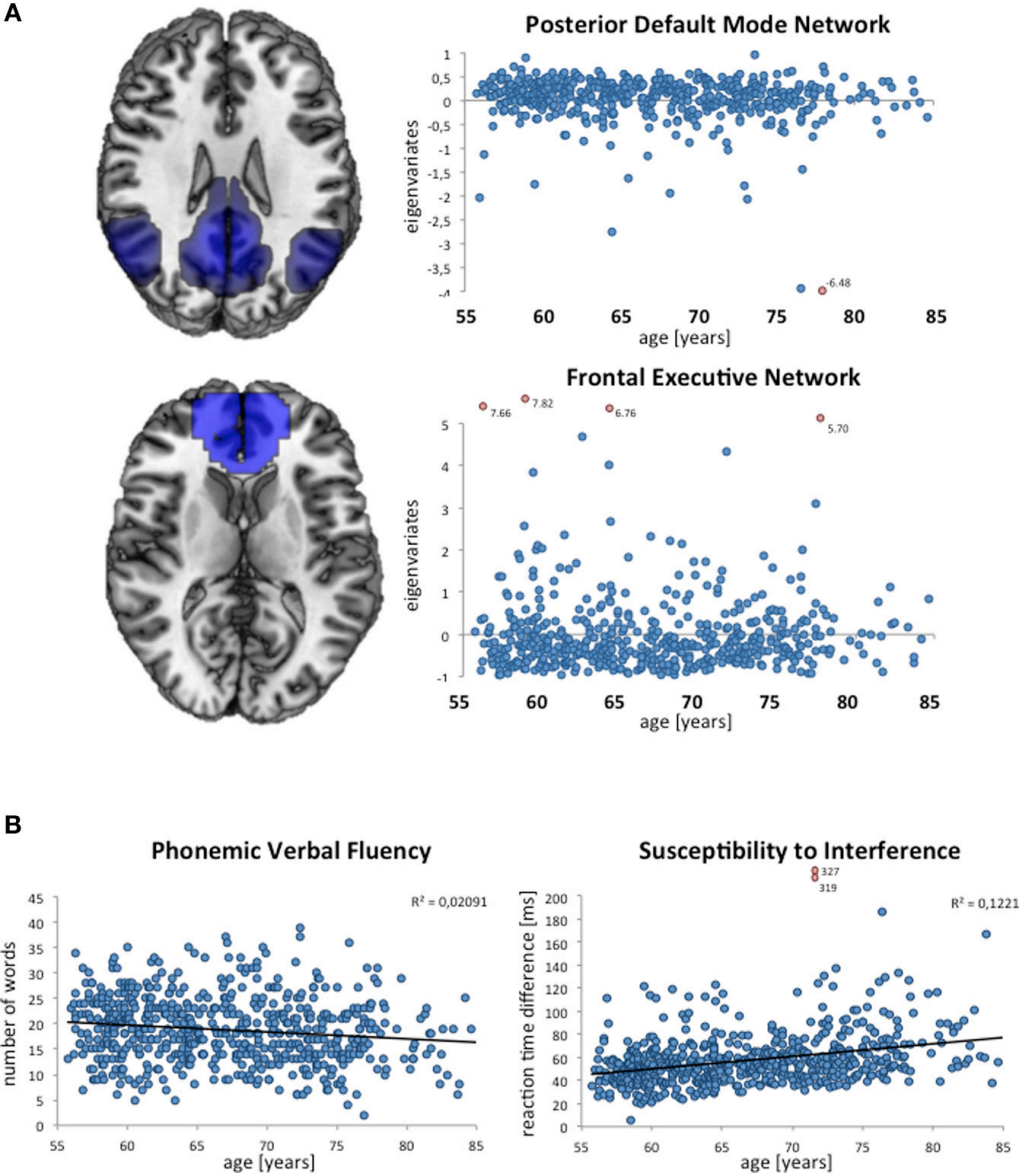
**Exemplary depictions of interindividual variability in phenotypes assessed in 1000BRAINS. (A)** Composition of resting-state networks and their variability with age, as expressed via the individual eigenvariates (ICA decomposition using MELODIC; Beckmann and Smith, 2004) within FSL (http://www.fmrib.ox.ac.uk/fsl), displayed for the posterior part of the default mode network and the frontal executive network. **(B**) Distribution of performance in verbal fluency (measured via the Regensburger Wortflüssigkeitstest, see Table 2) and susceptibility to interference (measured via the Farb-Wort-Interferenztest according to Stroop, see Table 2). Each blue dot indicates the resting-state network eigenvariate **(A)** and the raw test score as achieved in the respective neuropsychological test **(B)** for a given subject. Outliers are marked as red dots.

An example of variability in a specific brain phenotype is depicted in Figure 3A. Here, intersubject variability in the composition of the resting-state networks is demonstrated in terms of eigenvariates. Both the posterior default mode and the frontal executive network (as described by Smith et al., 2009) are present in all subjects. However, wide variation is observed between subjects in terms of the extent and involvement of the posterior cingulate and angular gyrus or medial frontal areas respectively, and thus the functional connectivity within these networks. The following text describes variables of interest in 1000BRAINS, i.e., variables that might explain some degree of the observed variability in brain phenotype.

#### Neuropsychological assessment

***Performance testing***. The neuropsychological performance tests applied in 1000BRAINS address a wide range of cognitive functions. These tests enable identification of cognitive functions that are differentially affected by aging, and assessment of the respective consequences on brain imaging parameters. The resulting neuropsychological profiles allow comparison of intra- and interindividual strengths and weaknesses, particularly in elderly subjects. Specific functional tests include assessment of performance in the domains of attention, memory, language skills, and executive functions. The presence of early signs of dementia is assessed using DemTect (Kalbe et al., 2004). This screening tool assesses verbal and working memory and word fluency performance as well as intellectual flexibility, and has a high sensitivity for the detection of early dementia and mild cognitive impairment. Total testing time is approximately 75–105 min, depending on subject performance. Table 2 provides an overview of all cognitive tests administered in 1000BRAINS.

**Table 2:** **Neuropsychological performance tests as administered in 1000BRAINS**.

**Test (References)**	**Functions**	**Description**
Trail making test A and B (taken from CERAD-Plus) (Morris et al., 1989)	A: Visual attention, processing speed	A: Connecting randomly arranged digits in an ascending order by drawing lines
	B: Concept shifting	B: Connecting alternately numbers and letters in ascending order
Alters-Konzentrations-Test (AKT)^*^ (Gatterer, 2008)	Selective attention	Cancel a target figure out of similar distractor figures
D2-R^**^ (Brickenkamp et al., 2010)	Selective attention	Cancel a target sign out of similar distractor signs
Verbaler Gedächtnistest (Lux et al., 2012)	Verbal episodic memory	Learning of a 15 words list in 5 trials, with free, cued direct, and delayed recall
Benton-Test (Benton et al., 2009)	Visual memory	Free recall of 20 figures
	Figural memory	
Block-Tapping-Test (Schelling, 1997)	Visual spatial working memory	Repeating a by trial increasing sequence of blocks on a board of 9 blocks, in equal and reverse order
Visual pattern (Jülich version) (similar to: Della Sala et al., 1997)	Visual working memory	Memorizing a matrix pattern of black and white squares in grids of increasing complexity
Fünf-Punkte-Test (Jülich version) (similar to: Regard et al., 1982)	Figural fluency	Drawing different designs by connecting 5 dots with lines
Subtest 3 (from Leistungsprüfsystem 50+) (Sturm et al., 1993)	Problem solving	Identifying (ir)regularity in a row of geometric figures
Zahlennachsprechen (from Nürnberger Alters-Inventar) (Oswald and Fleischmann, 1997)	Verbal working memory	Repeating a by trial increasing sequence of spoken numbers in equal and reverse order
Regensburger Wortflüssigkeitstest (Aschenbrenner et al., 2000)	Semantic/Phonemic verbal fluency	Producing words beginning with a given letter(s) or words from specific categories
Boston Naming Test (from CERAD; short form) (Morris et al., 1989)	Word retrieval	Naming ink drawings of objects with increasing difficulty
	Naming	
Farb-Wort-Interferenztest (Jülich version) (similar to: Stroop, 1935; Bäumler, 1985)	Visual attention	Reading words with color meaning; naming the color of colored boxes; naming the color of words with color meaning, printed in a different color
	Information processing speed	
	Susceptibility to interference	
Wortschatztest (Schmidt and Metzler, 1992)	Vocabulary	Identifying real words within a row of 5 pseudo-words
DemTect (Kalbe et al., 2004)	Verbal memory	
	Working memory	
	Word fluency	
	Intellectual flexibility	

Tests are listed in the order in which they are performed. CERAD, Consortium to Establish a Registry for Alzheimer's Disease;

* only used in subject older than 55 years of age,

** only used in subjects younger than 55 years of age.

Some cognitive tests are designed specifically for the testing of older subjects, i.e., 55 years and above, while others are designed to test subjects aged 18–55 years (cf. Table 2). This allows appropriate testing of specific cognitive functions in both elderly and younger subjects (i.e., those from the MultiGeneration Study).

Figure 3B illustrates the age distributions of the raw scores of two neuropsychological variables in the currently available 1000BRAINS dataset. As shown by these scatter plots, there is marked interindividual variability in these performance measures, reflecting the underlying heterogeneity of this population-based sample. Moreover, age effects seem to be present, in particular in the performance of the Color Word Interference Task (with older subjects taking longer to respond). Importantly, both the examplary neuroimaging and neuropsychological data illustrate a low number of outliers, indicating the successful implementation of our standardized approach.

***Self-report and observer-based ratings***. An important aspect of the process of aging is the subjective appraisal of aging and the importance of cognitive impairment in terms of well-being and quality of life (Reid and MacLullich, 2006; Jessen et al., 2010; Jeste et al., 2010). These factors are measured in 1000BRAINS using established psychometric questionnaires. These data will be analyzed within the context of the neuroscientific and neuropsychological data. Complementary analyses will focus on the normative aspects and ethical implications of the distinction between normal and pathological aging, and the normative and ethical implications of suspected or established dementia in terms of the affected person's standing within society (Sturma, 2011). A necessary condition for answering the question how mental and physical processes of normal aging differ from pathological changes is to establish criteria for the conception of “normality.” This will make any assumptions on this concept transparent and will render them open for argumentation and justification. It is of particular interest to clarify, which mental and behavioral expressions in early stages of dementia should still fall within the supposed range of normality. This particularly necessitates an understanding of how a person subjectively experiences her cognitive decline and the associated altered reactions from her social environment.

Data concerning personality, quality of life, daily activities, behavioral changes, psychiatric symptoms, handedness, and language skills are also obtained within 1000BRAINS. With the consent of the subject, ratings concerning changes in behavior are also obtained from family members. An overview of all self-rating and observer-based questionnaires used in 1000BRAINS is provided in Table 3.

**Table 3:** **Neuropsychological self- and observer-based rating scales as used in 1000BRAINS**.

**Scale (Reference)**	**Topic**	**Function/Dimension**
SF-36 Fragebogen zum Gesundheitszustand (Bullinger and Kirchberger, 1998)	Quality of life in relation to health	Self-report assessment of quality of life in relation to health and any kind of illness. Dimensions covered are: vitality, physical pain and functionality, physical and emotional role function, social functionality, and mental well-being
Nürnberger Alters-Alltagsaktivitäten-Skala^*^ (from Nürnberger Alters-Inventar) (Oswald and Fleischmann, 1997)	Daily activities in elderly	Rating of current instrumental and social abilities which allows estimating limitations in daily activities as experienced by the subjects
Nürnberger Lebensqualitäts-Fragebogen (from Nürnberger Alters Inventar) (Oswald and Fleischmann, 1997)	Quality of life in general	Assessment of general quality of life in elderly with dimensions contentedness, well-being, partnership, physical symptoms, and work performance
Beck-Depression-Inventory II (Hautzinger et al., 2006)	Indicators of depression	Self-report inventory assessing current symptoms of depression and their severity, covering affective, and somatic symptoms
Jülich Psychiatric Screening Questionnaire (JPS)	Axis I and II Disorders (DSM-IV)	Questionnaire on the psychological status in terms of major mental and personality disorders
Edinburgh Handedness Inventory (Oldfield, 1971)	Handedness	Preference in using right or left hand, eye and foot
Freiburger Persönlichkeitsinventar (FPI-R) (Fahrenberg et al., 2010)	Personality	Self-report instrument to assess personality traits on 12 subscales: life satisfaction, social orientation, performance orientation, shyness, irritability, aggression, stress, physical complaints, health concerns, openness; and 2 secondary factors (cf. Eysenck): extraversion, emotionality (neuroticism)
Jülicher Inventar zu frontalem Verhalten (modified from Frontal behavioral inventory) (similar to: Kertesz et al., 1997)	Personality and behavioral changes	Possible behavioral changes related to a decrease of frontal brain tissue are investigated
Jülicher Fragebogen zur Ermittlung von Sprachkenntnissen (modified from Language experience and proficiency questionnaire) (similar to: Marian et al., 2007)	Language skills	Assessment of language skills in mother tongue as well as any foreign language(s)
Jülicher Kurzfragebogen zur aktuellen Beanspruchung (modified from Kurzfragebogen zur aktuellen Beanspruchung) (Müller and Basler, 1993)	Current stress level	Questionnaire on the evaluation of the current stress level before and after testing
Jülicher Fragebogen zu Subjektiven Kognitiven Beeinträchtigungen I & II (JFSKB-I & JFSKB-II)	Subjective cognitive impairment	Questionnaires for obtaining subjective impairment in several areas of cognitive functioning (absolute and comparative)
Jülicher Fragebogen zur Alzheimer Krankheit und Demenz	Alzheimer's disease and dementia	Questionnaire on the evaluation of and coping with Alzheimer's disease and dementia

* Only used in subject older than 55 years of age.

#### Motor assessment

***Performance testing***. Motor performance is assessed using tests that evaluate motor functions that decline in old age. These include uni- and bimanual fine- and gross motor skills for proximal and distal upper limb function, as well as motor sequence learning. Lower limb and gait performance is assessed using various tests of gait. These account for dual task performance by requiring a combination of motor and/or cognitive task performance while walking. Stability of stance and balancing capabilities are assessed on an oscillatory platform, in a test which requires the subject to counterbalance sudden platform displacement. An overview of all 1000BRAINS motor performance tests is provided in Table 4.

**Table 4:** **Motor assessment as administered in 1000BRAINS**.

**Test (Reference)**	**Functions/Parameters**	**Description**
**PERFORMANCE TESTS**		
Finger Pointing test (adopted from CAPSIT-PD) (Defer et al., 1999)	Fine motor coordination (speed and accuracy)	Moving index finger 10 times between two target buttons, which are 30 cm apart from each other, at maximum pace
Finger Tapping test (adopted from Halstead, 1947)	Manual dexterity, fine motor coordination (rate, regularity)	Tapping index fingers
		1. With right/left at internal pace
		2. With right/left at maximum pace
		3. Alternating between right and left index fingers with increasing and decreasing pace
Motor Sequences test (adopted from Luria, 1973)	(Uni- /Bimanual Motor Sequence Learning)	Series of hand movements
		1. Left/right hand: fist, edge, palm
		2. Bimanual: alternating fist, palm
		3. Bimanual: alternating dorso-ventral hand flaps with elevated and flexed arms
Multiple Tasks Gait test (Jülich version) (similar to: Haggard et al., 2000; Bloem et al., 2001)	Standard gait parameters (speed, step frequency, cadence)	Walking for 25 m
		1. Free at internal pace
	Multiple tasks performance (Motor/Cognitive-tasks)	2. With loaded tray (glass and cup (saucer) filled with water)
		3. 2 + spatial imagination task (determining whether position of hour and minute hands are on the same or on different sides of the clock face, for various given times)
		4. 2 + verbal task (deciding if given words contain letters A and R)
Posturomed Oscillatory Platform (Posturomed®, HAIDER BIOSWING Weiden, Germany) (Müller et al., 2004; Boeer et al., 2010)	Ability to control balance on a displaced oscillatory platform	Two-legged stance counterbalancing of platform movement following standardized sudden platform displacements toward left, right, and rear
	Vestibular-Cerebellar-Proprioceptive functions	
**SELF- AND OBSERVER-BASED RATINGS**		
EPESE Score (Guralnik et al., 1994)	Lower extremity function, predictor of mortality estimation and nursing home admission	Total score of tests of balance, time to walk 8 foot, and time to rise from a chair 5 times
FTSS Score (Buatois et al., 2008)	Postural stability, prediction of risk of falls	Time to rise from a chair 5 times without arm/hand assistance
(Semi-) Tandem Stance (from Berg Balance Scale) (Berg et al., 1989)	Postural stability	Both feet placed along a single straight line (toes touching heel in tandem stance, not touching in semitandem stance)
UPDRS Part III (Martínez-Martín et al., 1994)	Detection of (subclinical) Parkinson motor symptoms	Motor examination with rating of gait, stance, and hand/arm/leg movements
Activities-specific Balance Confidence Scale (ABC) (Powell and Myers, 1995)	Balance self-confidence	Subjective assessment of balance confidence in different daily activities

CAPSIT-PD, Core Assessment Program for Surgical Interventional Therapies; EPESE, Established Populations for the Epidemiologic Study of the Elderly; FTSS, Five Time Sit to Stand; UPDRS, Unified Parkinson Disease Rating Scale.

***Self-report and observer-based ratings***. Gait and stance capabilities are also evaluated using observer-rated tests. For the detection of subclinical Parkinsonism, part III of the *Unified Parkinson Disease Rating Scale* from routine clinical practice is administered (Martínez-Martín et al., 1994). In addition, subjects are asked to complete a self-report questionnaire concerning self-confidence in maintaining balance in a variety of daily activities. An overview of the scores and ratings is provided in Table 4.

#### Laboratory, genetic, epigenetic, and expression data

For each HNR and HNR-MultiGeneration subject, blood samples are drawn at the HNR study appointment in Essen. Within 1000BRAINS, measures of interest are those that are likely to influence the structure and function of the aging brain. These include hormones, metals, vitamins, lipids, dietary minerals as well as cytokines (Moffat, 2005; Brewer, 2012; Joshi and Praticò, 2012; Lachner et al., 2012; Maggio et al., 2012). Furthermore, cellular or molecular markers of aging will be of interest (Lopéz-Otín et al., 2013), particularly if the situation in the peripheral blood reflects the situation in the brain.

**Figure 4:**
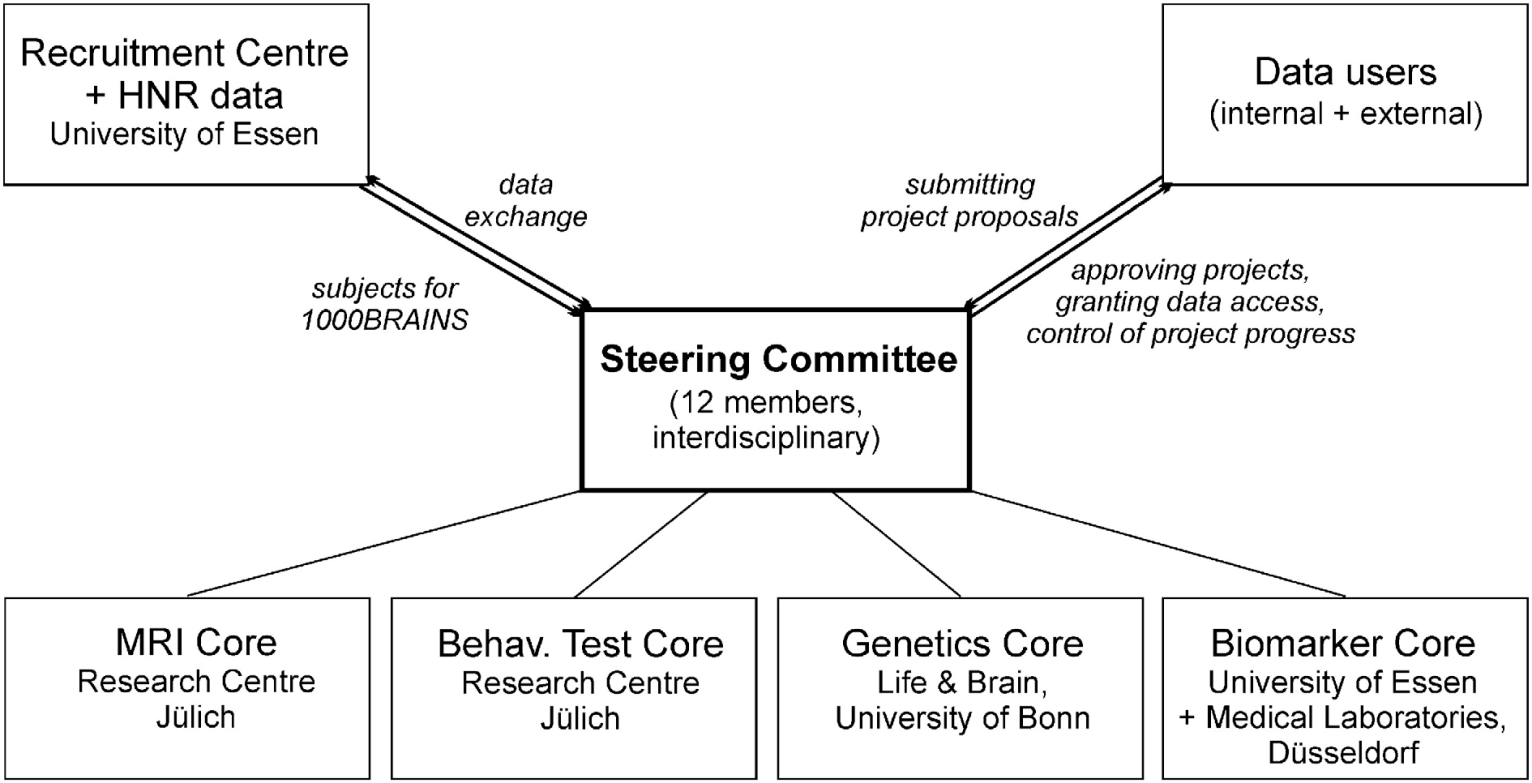
**Management and organizational structure of the 1000BRAINS study**.

For the genetic and epigenetic analyses, EDTA-anticoagulated blood samples are stored separately as aliquots at −80°C. In a first step, genetic analyses within 1000BRAINS will focus on the identification of common genetic factors with an influence on brain aging. This refers in particular to the most abundant form of variation in the human genome, i.e., single nucleotide polymorphisms (SNPs). A systematic genome-wide search for SNPs with an influence on brain aging will be performed (genome-wide association study). This will involve microarrays that enable the parallel genotyping of millions of SNPs. Previous research has identified a number of SNPs with a structural or functional influence on brain phenotypes (Esslinger et al., 2009; Erk et al., 2010; Bis et al., 2012; Stein et al., 2012). However, most studies have focused on the effects of disease-associated genetic variants (for review: Meyer-Lindenberg and Weinberger, 2006; Meyer-Lindenberg and Zink, 2007; Meyer-Lindenberg, 2010), and scant data are available concerning genetic factors with an influence on variability in brain phenotypes. Genotyping is performed at the Life & Brain Center of the University of Bonn (Figure 4), using Illumina's microarrays of the Omni series, which encompasses up to 1.92 million SNPs per individual. Use of genome-wide SNP data also will allow the application of methods which attempt to account for the pronounced polygenicity of many complex phenotypes. Polygenic score analyses evaluate whether common variants have an important role on normal aging *en masse* through direct testing of the classic theory of polygenic inheritance (e.g., International Schizophrenia Consortium, 2009). Another widely used approach that will be applied in 1000BRAINS is pathway analysis. The aim of this approach is to identify whether extended lists of SNPs with association signals are enriched in defined biological pathways.

Besides these systematic, genome-wide searches for genetic factors with an influence on phenotypes of interest within 1000BRAINS, analyses of individual candidate genes or candidate gene systems will be performed. Of particular interest is the question of the extent to which common genetic risk factors in genes previously implicated in brain disorders, e.g., neurodegenerative or neuropsychiatric disorders, influence the observed variability in brain aging.

In addition, epigenetic studies focusing on DNA methylation and studies of gene expression will be performed. Future use of exome- or genome-wide sequencing might also be considered within 1000BRAINS.

### Data storage, management, and access

All data are stored in a pseudonymized manner in accordance with current data safety and protection guidelines. To optimize management of the extensive neuroimaging-, neuropsychological-, and genetic data sets generated in 1000BRAINS, the LORIS (Longitudinal Online Research and Imaging System) is being used. LORIS is a neuroinformatics research database system developed by the Montreal Neurological Institute at McGill University, Montreal, Canada (http://www.loris.ca; Das et al., 2012). LORIS is based on a MySQL database server and offers a web-based user interface, which renders it particularly suitable for multi-center trials (e.g., NIHPD—US study on normal child brain development, IBIS—US study on autism, GUSTO—large birth cohort of the University of Singapore). To ensure compliance with Germany's strict data security and handling guidelines, LORIS has been installed on a separate server at the Institute of Neuroscience and Medicine, Research Centre Jülich, Germany, with no possibility of external access. An important advantage of LORIS is its capability for the automatized quality control of entered data. The double-data entry option ensures that incorrect entries are detected.

Access to 1000BRAINS data by researchers will be granted on the basis of the scientific value of the proposed research project (Figure 4). A steering committee comprised of the principal investigators of 1000BRAINS has been established. This committee includes experts in neuroscience, epidemiology, and genetics. The steering committee will decide upon the acceptance of research proposals using defined data usage guidelines. Upon completion of the respective research project, the investigators will be required to forward all results to the 1000BRAINS group at the Research Centre Jülich, where they will be archived. Proposals for collaborative projects should be directed to k.amunts@fz-juelich.de or s.caspers@fz-juelich.de.

## Discussion

### Study design: strengths and limitations

1000BRAINS has been designed to investigate variability in human brain aging. This is facilitated by two key aspects of the study: (i) use of the extensive dataset of the unique population-based HNR Study, which has been obtained through the 10 year longitudinal evaluation of cardiovascular, life style, and socioeconomic factors; and (ii) investigation of a wide range of potential influencing factors and their interactions, using a multi-modal approach comprising neuroimaging, behavioral and cognitive testing, genetics, and blood parameters. The interactions between genetic and lifestyle factors can be regarded as being particularly relevant. It could be assumed that age-related changes are amplified or counteracted by given lifestyle variables (Watt, 2014). The combination of the 1000BRAINS data with the surveys on exercise, dietary habits, sleep, and social environment of the subjects, which are available via the database of the HNR Study (Schmermund et al., 2002), provides the opportunity to investigate into these interactions. This will broaden the understanding of the role of genes and lifestyle on the process of aging.

Age-related changes in brain structure and function might be subtle. Large samples are therefore required to detect small, but nonetheless relevant, effects. This is also the case for genetic influences. Recent studies on different brain phenotypes have reported notable genetic effects, even for genetic factors with a low penetrance (Bis et al., 2012; Stein et al., 2012). In terms of variability in human brain aging, each individual genetic factor might explain only a small proportion of the striking variability observed in aging brain phenotypes. To ensure adequate sample sizes, recent investigations have pooled samples from a number of studies (e.g., Stein et al., 2012). While this generates very large samples, such efforts raise potential difficulties in terms of data heterogeneity. An advantage of 1000BRAINS is that its well-characterized sample is being examined using the same equipment and research instruments. Reducing technical and methodical variability is a crucial prerequisite for the reliable investigation of biological interindividual variability.

As in other *in-vivo* neuroimaging studies, analysis of the variability of structural, functional, and connectional phenotypes of the aging brain within 1000BRAINS is limited to millimeter resolution. To integrate neuroanatomical information at the microscopic level into the 1000BRAINS analyses, data from the JuBrain Cytoarchitectonic Atlas (http://www.jubrain.fz-juelich.de) will be included. This atlas classifies the cortex and the subcortical structures on the basis of microstructural criteria. Precise anatomical definition of structurally-functionally relevant subunits of the cortex offers several advantages. Firstly, it allows anatomically and neurobiologically informed interpretation of the neuroimaging data (Zilles and Amunts, 2010; Caspers et al., 2013). Secondly, it increases the methodical homogeneity of the data by defining these modules in a standardized manner. Thirdly, it reduces technical variance. This in turn allows more specific characterization of factors which influence variability in human brain aging.

### Ethical considerations

1000BRAINS has established a standardized procedure for the detection and reporting of incidental findings. This combines expert radiological review, discussion within an internal disease advisory board, and the provision of regular advice by experts in neuroethics. At the time of writing, the issue of incidental findings is a matter of intense debate within the neuroimaging community (Illes et al., 2004a; Booth et al., 2010; Lanzerath et al., 2013). It is also an important issue for ethical review boards and for the field of neuroethics in general (Illes et al., 2004b; Illes and Chin, 2008; Wolf et al., 2008). Since incidental findings are prevalent in the general population (2–8%; Illes et al., 2004a; Vernooij et al., 2007), they must be anticipated in a large-scale neuroimaging project, particularly one involving the investigation of elderly subjects using higher MR field strengths and higher resolution of the structural sequences (Morris et al., 2009). The issue of how to manage incidental findings within neuroimaging research involves consideration of the subject's right to self-determined knowledge, a physician's duty and professional ethical code with respect to informing an individual of the presence of a potentially serious condition and the offering of appropriate management, and the interest of society and the national economy in avoiding the cost of unnecessary clinical examinations. A recent report concerning experience in the SHIP found that the majority of subjects expressed a wish to be informed of any relevant finding (Schmidt et al., 2013). The approach used within 1000BRAINS is designed to take the competing interests of physicians, researchers and participants into account. In doing so, 1000BRAINS adheres to the current recommendations for the ethical management of incidental and secondary findings of the Presidential Commission for the Study of Bioethical Issues (http://www.bioethics.gov).

1000BRAINS also aims to elucidate the subject's personal experience of the aging process and how this correlates with objective measures of aging, i.e., those related to variability in brain structure and function and performance. The aging process is also perceptible within society as a whole. Distinguishing specific aspects of the aging process within individuals from the overall, normative aspects of aging is therefore of interest (McMullin, 2000), since this determines how the elderly are perceived and accepted within society, and which ethical issues should be considered, e.g., when working with individuals with dementia (Sturma, 2011). 1000BRAINS approaches these questions by including self-reported appraisal of the aging process in relation to performance, as well as life-style- and environmental factors.

### 1000BRAINS in relation to other epidemiological cohort and large-scale imaging studies

Over recent years, several large-scale neuroimaging projects have been established for the investigation of diverse hypotheses. Most have focused on studying changes in the brain in relation to disease. The following discussion focuses on those aspects of 1000BRAINS that are novel in relation to other on-going neuroimaging projects.

The Rotterdam Study (Hofman et al., 1991) commenced in 1990 with the investigation of subjects aged 55 years and above. In 2000 and 2006, the cohort was extended with the inclusion of subjects aged 45 years and above. The main focus of the Rotterdam study is the identification of causes of common diseases in the elderly. In terms of neurology, the major foci of the study are neurodegenerative and cerebrovascular diseases, different types of dementia, Parkinson's disease, and stroke (Hofman et al., 2007, 2009, 2011). The relation between changes in brain structure and function and relevant disease-related risk factors is addressed using 1.5T MR imaging (de Groot et al., 2001; Ikram et al., 2012). CASCADE (Cardiovascular Determinants of Dementia; Launer et al., 2000) is a collaboration of various established cohort studies. Here, short- and long-term cardiovascular causes of dementia are assessed within the pooled dataset of the included cohorts—with a focus on white matter lesions and gray matter structure—using 1.0T MRI imaging. The ADNI project (Alzheimer's Disease Neuroimaging Initiative; Mueller et al., 2005; Weiner et al., 2010, 2012) is a multi-center study which aims to identify differences between healthy elderly subjects, subjects with mild cognitive impairment, and patients with Alzheimer's disease. The major focus of the ADNI neuroimaging protocol is brain structure, with investigation of structural and functional connectivity. Being multi-centric, ADNI involves different MR scanners from different vendors, all of which have different field strengths (1.5 and 3T).

Whereas the aforementioned studies focus on neuroimaging, the research aims of SHIP (Völzke et al., 2011) are very broad. This study commenced in 1997 and covers a wide range of diseases and related risk factors in subjects aged 20–70 years. The neuroimaging aspects of the SHIP whole-body MR imaging protocol at 1.5T focus on structural sequences (Hegenscheid et al., 2009).

The ongoing Human Connectome Project (HCP; Toga et al., 2012; Van Essen et al., 2012) is a large scale neuroimaging project. This aims to study structural and functional brain connectivity in sibships aged 20–35 years. The study protocol is focused on advanced MR imaging at 3T and, in some cases, 7T, and involves specially developed and optimized imaging sequences for studying structural connectivity in relation to behavioral and genetic data.

In contrast to these projects, 1000BRAINS focuses on aging of the brain in elderly subjects. 1000BRAINS has been designed to characterize interindividual variability in the aging process. 1000BRAINS is unique in combining neuroimaging for the detailed assessment of brain structure, function and connectivity in the brain of older subjects with analysis of a variety of environmental-, genetic-, life-style-, and behavioral factors. By building upon the wealth of longitudinal data from the HNR Study, the scope of 1000BRAINS is widened, thus allowing assessment of the trajectories of potential influencing factors over a period of 10 years.

## Conclusions

1000BRAINS is based upon the population-based cohort of the HNR study, which reached its 10-year follow-up stage in 2011 (Erbel et al., 2012). Use of the longitudinal HNR data and cohorts will allow identification of factors with a potential influence on brain aging, e.g., cardiovascular- and life style factors. The systematic assessment of variability in brain structure and function in the general population, with a major focus on the brain of elderly subjects, will provide a neuroscientific basis for understanding alterations in the diseased brain across a spectrum that comprises normal variability, disturbed functioning, and manifest disease.

### Conflict of interest statement

The authors declare that the research was conducted in the absence of any commercial or financial relationships that could be construed as a potential conflict of interest.
